# Supplementary material for: Quantifying the under-reporting of uncorrelated longitudal data: the genital warts example
Source: BMC Med Res Methodol. 2021 Jan 6;21:6. doi: 10.1186/s12874-020-01188-4 (PMC7789373; doi:10.1186/s12874-020-01188-4)
Supplement: Supplementary file 1 — Additional file 1 Additional tables and R code to reproduce the analyses. [file 12874_2020_1188_MOESM1_ESM.pdf]

SUPPLEMENTARY MATERIAL

Accompanying the manuscript:

Quantifying the under-reporting of uncorrelated longitudinal data: The genital warts example

David Moriña*
Department of Econometrics, Statistics and Applied Economics, Riskcenter-IREA, Universitat de Barcelona (UB)
Centre de Recerca Matemàtica
E-mail: dmorina@ub.edu

Amanda Fernández-Fontelo
Chair of Statistics, School of Business and Economics, Humboldt-Universität zu Berlin
E-mail: fernanda@hu-berlin.de

Alejandra Cabaña
Departament de Matemàtiques, Universitat Autònoma de Barcelona, Barcelona Graduate School of Mathematics (BGSMath)
E-mail: acabana@mat.uab.cat

Pedro Puig
Departament de Matemàtiques, Universitat Autònoma de Barcelona, Barcelona Graduate School of Mathematics (BGSMath)
E-mail: ppuig@mat.uab.cat

Laura Monfil
Unit of Infections and Cancer - Information and Interventions (UNIC - I&I), Cancer Epidemiology Research Program (CERP), Catalan Institute of Oncology (ICO)-IDIBELL
E-mail: lmonfil@iconcologia.net

Maria Brotons
Unit of Infections and Cancer - Information and Interventions (UNIC - I&I), Cancer Epidemiology Research Program (CERP), Catalan Institute of Oncology (ICO)-IDIBELL
E-mail: mbrotons@iconcologia.net

Mireia Diaz
Unit of Infections and Cancer - Information and Interventions (UNIC - I&I), Cancer Epidemiology Research Program (CERP), Catalan Institute of Oncology (ICO)-IDIBELL
E-mail: mireia@iconcologia.net

*dmorina@ub.edu

1. Table S1. Under-reporting magnitude for each subpopulation by year

Year	Sex	Age	SIDIAP incidence (registered)	SIDIAP incidence (reconstructed)	Difference (%)
**2009**	**Females**	**15-29**	14.67	19.63	33.76%
**2010**			17.78	22.48	26.41%
**2011**			18.74	23.14	23.52%
**2012**			19.04	23.51	23.46%
**2013**			20.66	23.68	14.59%
**2014**			21.57	24.81	15.04%
**2015**			19.92	24.61	23.56%
**2016**			19.63	22.04	12.27%
**Average**			19.00	22.99	20.97%
**2009**		**30-94**	2.66	3.56	33.76%
**2010**			3.29	4.40	33.76%
**2011**			3.41	4.57	33.76%
**2012**			3.66	4.89	33.76%
**2013**			4.26	5.70	33.76%
**2014**			4.61	5.99	30.10%
**2015**			4.76	5.38	13.04%
**2016**			4.91	4.91	0.00%
**Average**			3.95	4.93	24.85%
**2009**	**Males**	**15-29**	11.50	15.38	33.76%
**2010**			14.23	18.52	30.13%
**2011**			15.36	20.55	33.76%
**2012**			17.59	21.04	19.63%
**2013**			21.59	23.48	8.76%
**2014**			22.95	22.95	0.00%
**2015**			22.40	22.79	1.75%
**2016**			21.20	21.63	2.02%
**Average**			18.35	20.79	13.30%
**2009**		**30-94**	3.49	4.67	33.76%
**2010**			4.50	6.02	33.76%
**2011**			4.76	6.37	33.76%
**2012**			4.93	6.59	33.76%
**2013**			6.63	8.87	33.76%
**2014**			7.04	8.90	26.46%
**2015**			7.70	7.87	2.24%
**2016**			7.86	7.86	0.00%
**Average**			5.86	7.14	21.83%

2. Table S2. Estimated probabilities of membership (females)

Age	Underreported	Non underreported	Age	Underreported	Non underreported
	1,000	0,000		0,960	0,040
	0,999	0,001		0,954	0,046
	0,932	0,068		0,934	0,066
	1,000	0,000		0,939	0,061
	0,995	0,005		0,915	0,085
	0,962	0,038		0,925	0,075
	0,974	0,026		0,921	0,079
	0,996	0,004		0,946	0,054
	0,997	0,003		0,920	0,080
	0,998	0,002		0,939	0,061
	0,961	0,039		0,942	0,058
	1,000	0,000		0,934	0,066
	0,924	0,076		0,905	0,095
	0,000	1,000		0,896	0,104
	0,986	0,014		0,893	0,107
	0,987	0,013		0,914	0,086
	0,779	0,221		0,915	0,085
	0,999	0,001		0,915	0,085
	0,016	0,984		0,914	0,086
	0,999	0,001		0,902	0,098
	0,995	0,005		0,899	0,101
	0,969	0,031		0,914	0,086
	0,971	0,029		0,904	0,096
**15-29**	0,998	0,002	**30-94**	0,910	0,090
	0,999	0,001		0,899	0,101
	0,945	0,055		0,898	0,102
	0,018	0,982		0,879	0,121
	0,416	0,584		0,881	0,119
	0,000	1,000		0,865	0,135
	0,856	0,144		0,889	0,111
	0,930	0,070		0,893	0,107
	0,999	0,001		0,886	0,114
	0,628	0,372		0,847	0,153
	0,978	0,022		0,869	0,131
	0,998	0,002		0,871	0,129
	1,000	0,000		0,885	0,115
	0,999	0,001		0,877	0,123
	0,987	0,013		0,855	0,145
	0,988	0,012		0,854	0,146
	0,989	0,011		0,856	0,144
	0,017	0,983		0,806	0,194
	0,998	0,002		0,829	0,171
	0,000	1,000		0,813	0,187
	0,982	0,018		0,854	0,146
	0,983	0,017		0,837	0,163
	0,038	0,962		0,793	0,207
	0,771	0,229		0,815	0,185

1,000	0,000	0,838	0,162
0,039	0,961	0,793	0,207
0,948	0,052	0,783	0,217
0,003	0,997	0,796	0,204
0,001	0,999	0,692	0,308
0,998	0,002	0,767	0,233
0,993	0,007	0,774	0,226
0,006	0,994	0,714	0,286
0,998	0,002	0,775	0,225
0,997	0,003	0,709	0,291
0,000	1,000	0,713	0,287
0,244	0,756	0,745	0,255
0,975	0,025	0,764	0,236
0,000	1,000	0,703	0,297
0,000	1,000	0,688	0,312
0,088	0,912	0,699	0,301
0,960	0,040	0,638	0,362
0,485	0,515	0,526	0,474
0,766	0,234	0,659	0,341
0,122	0,878	0,599	0,401
0,997	0,003	0,676	0,324
0,952	0,048	0,675	0,325
0,000	1,000	0,450	0,550
0,947	0,053	0,554	0,446
0,617	0,383	0,652	0,348
0,957	0,043	0,574	0,426
0,030	0,970	0,549	0,451
0,017	0,983	0,579	0,421
0,948	0,052	0,524	0,476
0,845	0,155	0,394	0,606
0,969	0,031	0,430	0,570
0,000	1,000	0,479	0,521
0,949	0,051	0,532	0,468
0,985	0,015	0,485	0,515
0,996	0,004	0,423	0,577
0,872	0,128	0,350	0,650
0,899	0,101	0,443	0,557
0,928	0,072	0,432	0,568
0,238	0,762	0,356	0,644
0,986	0,014	0,295	0,705
0,135	0,865	0,250	0,750
0,012	0,988	0,346	0,654
0,001	0,999	0,261	0,739
0,288	0,712	0,321	0,679
0,997	0,003	0,349	0,651
0,321	0,679	0,214	0,786
0,584	0,416	0,246	0,754
0,008	0,992	0,225	0,775
0,994	0,006	0,299	0,701

3. Table S3. Estimated probabilities of membership (males)

Age	Underreported	Non underreported	Age	Underreported	Non underreported
	0,999	0,001		0,968	0,032
	0,999	0,001		0,966	0,034
	0,992	0,008		0,951	0,049
	0,998	0,002		0,950	0,050
	0,999	0,001		0,937	0,063
	0,999	0,001		0,947	0,053
	0,986	0,014		0,905	0,095
	0,997	0,003		0,949	0,051
	0,997	0,003		0,935	0,065
	0,993	0,007		0,943	0,057
	0,918	0,082		0,951	0,049
	0,999	0,001		0,955	0,045
	0,998	0,002		0,937	0,063
	0,997	0,003		0,936	0,064
	0,989	0,011		0,914	0,086
	0,999	0,001		0,924	0,076
	0,567	0,433		0,906	0,094
	0,998	0,002		0,915	0,085
	0,985	0,015		0,933	0,067
	0,991	0,009		0,938	0,062
	0,999	0,001		0,907	0,093
	0,992	0,008		0,921	0,079
	0,465	0,535		0,913	0,087
**15-29**	0,999	0,001	**30-94**	0,931	0,069
	0,997	0,003		0,917	0,083
	0,986	0,014		0,904	0,096
	0,995	0,005		0,914	0,086
	1,000	0,000		0,924	0,076
	0,558	0,442		0,822	0,178
	0,986	0,014		0,904	0,096
	0,951	0,049		0,883	0,117
	0,999	0,001		0,925	0,075
	0,709	0,291		0,886	0,114
	0,723	0,277		0,915	0,085
	0,992	0,008		0,900	0,100
	0,999	0,001		0,921	0,079
	0,999	0,001		0,899	0,101
	0,988	0,012		0,892	0,108
	0,508	0,492		0,861	0,139
	1,000	0,000		0,884	0,116
	0,007	0,993		0,852	0,148
	0,145	0,855		0,878	0,122
	0,048	0,952		0,886	0,114
	0,964	0,036		0,903	0,097
	0,998	0,002		0,863	0,137
	0,000	1,000		0,834	0,166
	0,941	0,059		0,891	0,109

0,998	0,002	0,893	0,107
0,000	1,000	0,567	0,433
0,988	0,012	0,795	0,205
0,001	0,999	0,763	0,237
0,000	1,000	0,725	0,275
0,000	1,000	0,606	0,394
0,002	0,998	0,808	0,192
0,000	1,000	0,548	0,452
0,718	0,282	0,799	0,201
0,910	0,090	0,797	0,203
0,000	1,000	0,518	0,482
0,006	0,994	0,783	0,217
0,998	0,002	0,830	0,170
0,306	0,694	0,735	0,265
0,007	0,993	0,638	0,362
0,070	0,930	0,520	0,480
0,000	1,000	0,717	0,283
0,007	0,993	0,272	0,728
0,000	1,000	0,715	0,285
0,000	1,000	0,619	0,381
0,443	0,557	0,719	0,281
0,000	1,000	0,569	0,431
0,000	1,000	0,182	0,818
0,167	0,833	0,616	0,384
0,003	0,997	0,634	0,366
0,038	0,962	0,390	0,610
0,005	0,995	0,395	0,605
0,000	1,000	0,208	0,792
0,223	0,777	0,456	0,544
0,000	1,000	0,286	0,714
0,000	1,000	0,451	0,549
0,000	1,000	0,185	0,815
0,996	0,004	0,547	0,453
0,013	0,987	0,223	0,777
0,000	1,000	0,280	0,720
0,000	1,000	0,252	0,748
0,000	1,000	0,441	0,559
0,983	0,017	0,237	0,763
0,000	1,000	0,079	0,921
0,000	1,000	0,102	0,898
0,000	1,000	0,140	0,860
0,323	0,677	0,106	0,894
0,006	0,994	0,153	0,847
0,146	0,854	0,220	0,780
0,091	0,909	0,363	0,637
0,000	1,000	0,042	0,958
0,000	1,000	0,211	0,789
0,000	1,000	0,099	0,901
0,162	0,838	0,377	0,623

4. R code for the analysis

4.1. Log-likelihood function

```
llh <- function(pars, data, covars)
{
  loglik <- 0
  for (i in 1:length(data))
  {
    logit.w <- pars[1]+pars[2]*covars[i, 1]
    w       <- exp(logit.w)/(1+exp(logit.w))
    logit.q <- pars[11]
    q       <- exp(logit.q)/(1+exp(logit.q))
    loglik  <- loglik + log(w*dnorm(data[i], mean=(pars[3]+pars[4]*covars[i,
1]+pars[5]*covars[i, 2]+pars[6]*covars[i, 3]+pars[7]*covars[i, 4]+
                                                   pars[8]*covars[i, 5]+pars[9]*covars[i,
6]),
                                        sd=pars[10]) +
                                  (1-w)*dnorm(data[i], mean=(pars[3]+pars[4]*covars[i,
1]+pars[5]*covars[i, 2]+pars[6]*covars[i, 3]+pars[7]*covars[i, 4]+
                                                         pars[8]*covars[i,
5]+pars[9]*covars[i, 6])/q,
                                              sd=pars[10]/q))
  }
  return((-1)*loglik)
}
```

4.2. Main analysis

```
library(mixtools)

source("R/llh_covars.R") ### (-) log-likelihood function

### Read the data
load("Data/data_main.RData")

### Covariates matrix
t         <- rep(seq(1, 96), 4)/96
pr3$inter <- as.numeric(pr3$age)*as.numeric(pr3$sexe)
sint      <- sin(2*pi*t/3)
cost      <- cos(2*pi*t/3)
covars    <- cbind(t, pr3$age, pr3$sexe, pr3$inter, sint, cost)

### Direct estimation via mixtools
w0     <- 0.7
q0     <- 0.5
prova  <- regmixEM(pr3$incid, covars, lambda=c(w0, (1-w0)),
                   beta=matrix(c(mean(pr3$incid), 0, 0, 0, 0, 0, 0, mean(pr3$incid)/q0, 0, 0,
0, 0, 0 ,0), ncol=2, nrow=ncol(covars)+1),
                   sigma=c(sd(pr3$incid), sd(pr3$incid)/q0),
                   k=2, addintercept=TRUE, epsilon=1e-16, maxit=10000)

### Initial values for covariates from linear regression model
linmod <- lm(pr3$incid~covars[, 1]+covars[, 2]+covars[, 3]+covars[, 4]+covars[, 5]+covars[,
6])

### Estimates, standard errors and confidence intervals for w and q
max.llh <- nlm(f=llh, p=c(log(prova$lambda[which(prova$beta[1,]==min(prova$beta[1,]))]/(1-
prova$lambda[which(prova$beta[1,]==min(prova$beta[1,]))])),
                          -0.5, linmod$coefficients,
prova$sigma[which(prova$beta[1,]==min(prova$beta[1,]))],
                          log((prova$beta[1,which(prova$beta[1,]==min(prova$beta[1,]))]/
                               prova$beta[1,which(prova$beta[1,]==max(prova$beta[1,]))])/(1-
prova$beta[1,which(prova$beta[1,]==min(prova$beta[1,]))]/

prova$beta[1,which(prova$beta[1,]==max(prova$beta[1,]))]))),
               data=pr3$incid, covars=covars, hessian=TRUE)

q <- exp(max.llh$estimate[11])/(1+exp(max.llh$estimate[11]))

sigma        <- solve(max.llh$hessian)
lim.inf95_1  <- max.llh$estimate[1] - qnorm(0.975)*sqrt(diag(sigma))[1] #intercept w
lim.sup95_1  <- max.llh$estimate[1] + qnorm(0.975)*sqrt(diag(sigma))[1] #intercept w
lim.inf95_2  <- max.llh$estimate[2] - qnorm(0.975)*sqrt(diag(sigma))[2] #time w
lim.sup95_2  <- max.llh$estimate[2] + qnorm(0.975)*sqrt(diag(sigma))[2] #time w
```

```
lim.inf95_3  <- max.llh$estimate[3] - qnorm(0.975)*sqrt(diag(sigma))[3] #intercept mean
lim.sup95_3  <- max.llh$estimate[3] + qnorm(0.975)*sqrt(diag(sigma))[3] #intercept mean
lim.inf95_4  <- max.llh$estimate[4] - qnorm(0.975)*sqrt(diag(sigma))[4] #t
lim.sup95_4  <- max.llh$estimate[4] + qnorm(0.975)*sqrt(diag(sigma))[4] #t
lim.inf95_5  <- max.llh$estimate[5] - qnorm(0.975)*sqrt(diag(sigma))[5] #age
lim.sup95_5  <- max.llh$estimate[5] + qnorm(0.975)*sqrt(diag(sigma))[5] #age
lim.inf95_6  <- max.llh$estimate[6] - qnorm(0.975)*sqrt(diag(sigma))[6] #sex
lim.sup95_6  <- max.llh$estimate[6] + qnorm(0.975)*sqrt(diag(sigma))[6] #sex
lim.inf95_7  <- max.llh$estimate[7] - qnorm(0.975)*sqrt(diag(sigma))[7] #interaction age*sex
lim.sup95_7  <- max.llh$estimate[7] + qnorm(0.975)*sqrt(diag(sigma))[7] #interaction age*sex
lim.inf95_8  <- max.llh$estimate[8] - qnorm(0.975)*sqrt(diag(sigma))[8] #sin
lim.sup95_8  <- max.llh$estimate[8] + qnorm(0.975)*sqrt(diag(sigma))[8] #sin
lim.inf95_9  <- max.llh$estimate[9] - qnorm(0.975)*sqrt(diag(sigma))[9] #cos
lim.sup95_9  <- max.llh$estimate[9] + qnorm(0.975)*sqrt(diag(sigma))[9] #cos
lim.inf95_10 <- max.llh$estimate[10] - qnorm(0.975)*sqrt(diag(sigma))[10] #sd
lim.sup95_10 <- max.llh$estimate[10] + qnorm(0.975)*sqrt(diag(sigma))[10] #sd
lim.inf95_11 <- max.llh$estimate[11] - qnorm(0.975)*sqrt(diag(sigma))[11] #logit(q)
lim.sup95_11 <- max.llh$estimate[11] + qnorm(0.975)*sqrt(diag(sigma))[11] #logit(q)

### Confidence interval for q
exp(lim.inf95_11)/(1+exp(lim.inf95_11)); exp(lim.sup95_11)/(1+exp(lim.sup95_11))

### Reconstruction of the hidden processes
# a. Females, 15-29 years old
gw.women1 <- pr3[pr3$sexe==0 & pr3$age==0, ]
### Calculation of the posterior probabilities
post <- matrix(nrow=96, ncol=2)
w    <- vector()
for (i in 1:96)
{
  w[i] <-
exp(max.llh$estimate[1]+max.llh$estimate[2]*i/96)/(1+exp(max.llh$estimate[1]+max.llh$estimate[
2]*i/96))
  post[i, 1] <- w[i]*dnorm(gw.women1$incid[i],
mean=(max.llh$estimate[3]+max.llh$estimate[4]*i/96+max.llh$estimate[8]*covars[i, 5]+
                                                       max.llh$estimate[9]*covars[i, 6]),
sd=max.llh$estimate[10])/
    (w[i]*dnorm(gw.women1$incid[i],
mean=(max.llh$estimate[3]+max.llh$estimate[4]*i/96+max.llh$estimate[8]*covars[i, 5]+
                                            max.llh$estimate[9]*covars[i, 6]),
sd=max.llh$estimate[10])+
       (1-w[i])*dnorm(gw.women1$incid[i],
mean=(max.llh$estimate[3]+max.llh$estimate[4]*i/96+max.llh$estimate[8]*covars[i, 5]+
                                                  max.llh$estimate[9]*covars[i, 6])/q,
                   sd=max.llh$estimate[10]/q))
  post[i, 2] <- (1-w[i])*dnorm(gw.women1$incid[i],
mean=(max.llh$estimate[3]+max.llh$estimate[4]*i/96+max.llh$estimate[8]*covars[i, 5]+
                                                           max.llh$estimate[9]*covars[i,
6])/q, sd=max.llh$estimate[10]/q)/
    (w[i]*dnorm(gw.women1$incid[i],
mean=(max.llh$estimate[3]+max.llh$estimate[4]*i/96+max.llh$estimate[8]*covars[i, 5]+
                                            max.llh$estimate[9]*covars[i, 6]),
sd=max.llh$estimate[10])+
       (1-w[i])*dnorm(gw.women1$incid[i],
mean=(max.llh$estimate[3]+max.llh$estimate[4]*i/96+max.llh$estimate[8]*covars[i, 5]+
                                                  max.llh$estimate[9]*covars[i, 6])/q,
                      sd=max.llh$estimate[10]/q))
}
xrec <- ifelse(post[, 2] > 0.5, gw.women1$incid, gw.women1$incid/q)

mean(gw.women1$incid); mean(xrec)
(mean(xrec)-mean(gw.women1$incid))/mean(gw.women1$incid)*100

par(mfrow=c(2, 2))
gw.dones.ts <- ts(gw.women1$incid, start=c(2009, 1), end=c(2016, 12), freq=12)
ts.plot(gw.dones.ts, ylim=c(9, 32), ylab="Incidence x 100,000", main="Women 15-29 years old")
lines(seq(2009, 2016.99, 1/12), xrec, col="red", lty=2)

# b. Females, over 30 years old
gw.women2 <- pr3[pr3$sexe==0 & pr3$age==1, ]
### Calculation of the posterior probabilities
post <- matrix(nrow=96, ncol=2)
w    <- vector()
for (i in 1:96)
{
  w[i] <-
exp(max.llh$estimate[1]+max.llh$estimate[2]*i/96)/(1+exp(max.llh$estimate[1]+max.llh$estimate[
2]*i/96))
```

```
  post[i, 1] <- w[i]*dnorm(gw.women2$incid[i],
mean=(max.llh$estimate[3]+max.llh$estimate[4]*i/96+max.llh$estimate[5]+
                                                       max.llh$estimate[8]*covars[i,
5]+max.llh$estimate[9]*covars[i, 6]), sd=max.llh$estimate[10])/
     (w[i]*dnorm(gw.women2$incid[i],
mean=(max.llh$estimate[3]+max.llh$estimate[4]*i/96+max.llh$estimate[5]+max.llh$estimate[8]*cov
ars[i, 5]+
                                            max.llh$estimate[9]*covars[i, 6]),
sd=max.llh$estimate[10])+
       (1-w[i])*dnorm(gw.women2$incid[i],
mean=(max.llh$estimate[3]+max.llh$estimate[4]*i/96+max.llh$estimate[5]+max.llh$estimate[8]*cov
ars[i, 5]+
                                                  max.llh$estimate[9]*covars[i, 6])/q,
                      sd=max.llh$estimate[10]/q))
  post[i, 2] <- (1-w[i])*dnorm(gw.women2$incid[i],
mean=(max.llh$estimate[3]+max.llh$estimate[4]*i/96+max.llh$estimate[5]+max.llh$estimate[8]*cov
ars[i, 5]+
                                                           max.llh$estimate[9]*covars[i,
6])/q, sd=max.llh$estimate[10]/q)/
     (w[i]*dnorm(gw.women2$incid[i],
mean=(max.llh$estimate[3]+max.llh$estimate[4]*i/96+max.llh$estimate[5]+max.llh$estimate[8]*cov
ars[i, 5]+
                                            max.llh$estimate[9]*covars[i, 6]),
sd=max.llh$estimate[10])+
       (1-w[i])*dnorm(gw.women2$incid[i],
mean=(max.llh$estimate[3]+max.llh$estimate[4]*i/96+max.llh$estimate[5]+max.llh$estimate[8]*cov
ars[i, 5]+
                                                  max.llh$estimate[9]*covars[i, 6])/q,
                      sd=max.llh$estimate[10]/q))
}
xrec <- ifelse(post[,2] > 0.5, gw.women2$incid, gw.women2$incid/q)

mean(gw.women2$incid); mean(xrec)
(mean(xrec)-mean(gw.women2$incid))/mean(gw.women2$incid)*100

gw.dones.ts <- ts(gw.women2$incid, start=c(2009, 1), end=c(2016, 12), freq=12)
ts.plot(gw.dones.ts, ylim=c(1, 8), ylab="Incidence x 100,000", main="Women 30-94 years old")
lines(seq(2009, 2016.99, 1/12), xrec, col="red", lty=2)

# c. Males, 15-29 years old
gw.men1 <- pr3[pr3$sexe==1 & pr3$age==0, ]
### Calculation of the posterior probabilities
post <- matrix(nrow=96, ncol=2)
w    <- vector()
for (i in 1:96)
{
  w[i] <-
exp(max.llh$estimate[1]+max.llh$estimate[2]*i/96)/(1+exp(max.llh$estimate[1]+max.llh$estimate[
2]*i/96))
  post[i, 1] <- w[i]*dnorm(gw.men1$incid[i],
mean=(max.llh$estimate[3]+max.llh$estimate[4]*i/96+max.llh$estimate[6]+
                                                       max.llh$estimate[8]*covars[i,
5]+max.llh$estimate[9]*covars[i, 6]), sd=max.llh$estimate[10])/
    (w[i]*dnorm(gw.men1$incid[i],
mean=(max.llh$estimate[3]+max.llh$estimate[4]*i/96+max.llh$estimate[6]+max.llh$estimate[8]*cov
ars[i, 5]+
                                            max.llh$estimate[9]*covars[i, 6]),
sd=max.llh$estimate[10])+
       (1-w[i])*dnorm(gw.men1$incid[i],
mean=(max.llh$estimate[3]+max.llh$estimate[4]*i/96+max.llh$estimate[6]+max.llh$estimate[8]*cov
ars[i, 5]+
                                                  max.llh$estimate[9]*covars[i, 6])/q,
                      sd=max.llh$estimate[10]/q))
  post[i, 2] <- (1-w[i])*dnorm(gw.men1$incid[i],
mean=(max.llh$estimate[3]+max.llh$estimate[4]*i/96+max.llh$estimate[6]+max.llh$estimate[8]*cov
ars[i, 5]+
                                                           max.llh$estimate[9]*covars[i,
6])/q, sd=max.llh$estimate[10]/q)/
    (w[i]*dnorm(gw.men1$incid[i],
mean=(max.llh$estimate[3]+max.llh$estimate[4]*i/96+max.llh$estimate[6]+max.llh$estimate[8]*cov
ars[i, 5]+
                                            max.llh$estimate[9]*covars[i, 6]),
sd=max.llh$estimate[10])+
       (1-w[i])*dnorm(gw.men1$incid[i],
mean=(max.llh$estimate[3]+max.llh$estimate[4]*i/96+max.llh$estimate[6]+max.llh$estimate[8]*cov
ars[i, 5]+
                                                  max.llh$estimate[9]*covars[i, 6])/q,
                      sd=max.llh$estimate[10]/q))
```

```
}
xrec <- ifelse(post[,2] > 0.5, gw.men1$incid, gw.men1$incid/q)

mean(gw.men1$incid); mean(xrec)
(mean(xrec)-mean(gw.men1$incid))/mean(gw.men1$incid)*100

gw.homes.ts <- ts(gw.men1$incid, start=c(2009, 1), end=c(2016, 12), freq=12)
ts.plot(gw.homes.ts, ylim=c(4, 32), ylab="Incidence x 100,000", main="Men 15-29 years old")
lines(seq(2009, 2016.99, 1/12), xrec, col="red", lty=2)

# d. Males, 30-94 years old
gw.men2 <- pr3[pr3$sexe==1 & pr3$age==1, ]
### Calculation of the posterior probabilities
post <- matrix(nrow=96, ncol=2)
w    <- vector()
for (i in 1:96)
{
  w[i] <-
exp(max.llh$estimate[1]+max.llh$estimate[2]*i/96)/(1+exp(max.llh$estimate[1]+max.llh$estimate[
2]*i/96))
  post[i, 1] <- w[i]*dnorm(gw.men2$incid[i],
mean=(max.llh$estimate[3]+max.llh$estimate[4]*i/96+max.llh$estimate[5]+max.llh$estimate[6]+

max.llh$estimate[7]+max.llh$estimate[8]*covars[i, 5]+max.llh$estimate[9]*covars[i, 6]),
sd=max.llh$estimate[10])/
    (w[i]*dnorm(gw.men2$incid[i],
mean=(max.llh$estimate[3]+max.llh$estimate[4]*i/96+max.llh$estimate[5]+max.llh$estimate[6]+
                                          max.llh$estimate[7]+max.llh$estimate[8]*covars[i,
5]+max.llh$estimate[9]*covars[i, 6]), sd=max.llh$estimate[10])+
       (1-w[i])*dnorm(gw.men2$incid[i],
mean=(max.llh$estimate[3]+max.llh$estimate[4]*i/96+max.llh$estimate[5]+max.llh$estimate[6]+

max.llh$estimate[7]+max.llh$estimate[8]*covars[i, 5]+max.llh$estimate[9]*covars[i, 6])/q,
                      sd=max.llh$estimate[10]/q))
  post[i, 2] <- (1-w[i])*dnorm(gw.men2$incid[i],
mean=(max.llh$estimate[3]+max.llh$estimate[4]*i/96+max.llh$estimate[5]+max.llh$estimate[6]+

max.llh$estimate[7]+max.llh$estimate[8]*covars[i, 5]+max.llh$estimate[9]*covars[i, 6])/q,
sd=max.llh$estimate[10]/q)/
    (w[i]*dnorm(gw.men2$incid[i],
mean=(max.llh$estimate[3]+max.llh$estimate[4]*i/96+max.llh$estimate[5]+max.llh$estimate[6]+
                                          max.llh$estimate[7]+max.llh$estimate[8]*covars[i,
5]+max.llh$estimate[9]*covars[i, 6]), sd=max.llh$estimate[10])+
       (1-w[i])*dnorm(gw.men2$incid[i],
mean=(max.llh$estimate[3]+max.llh$estimate[4]*i/96+max.llh$estimate[5]+max.llh$estimate[6]+

max.llh$estimate[7]+max.llh$estimate[8]*covars[i, 5]+max.llh$estimate[9]*covars[i, 6])/q,
                      sd=max.llh$estimate[10]/q))
}
xrec <- ifelse(post[,2] > 0.5, gw.men2$incid, gw.men2$incid/q)

mean(gw.men2$incid); mean(xrec)
(mean(xrec)-mean(gw.men2$incid))/mean(gw.men2$incid)*100

gw.homes.ts <- ts(gw.men2$incid, start=c(2009, 1), end=c(2016, 12), freq=12)
ts.plot(gw.homes.ts, ylim=c(1, 12), ylab="Incidence x 100,000", main="Men 30-94 years old")
lines(seq(2009, 2016.99, 1/12), xrec, col="red", lty=2)
```

4.3. Validation

```
library(mixtools)
library(ggplot2)
library(gridExtra)

source("R/llh_covars.R") ### (-) log-likelihood function

### Read the data
load("Data/data_main.RData")

### Covariates matrix
t         <- rep(seq(1, 96), 4)/96
pr3$inter <- as.numeric(pr3$age)*as.numeric(pr3$sexe)
sint      <- sin(2*pi*t/3)
cost      <- cos(2*pi*t/3)
covars    <- cbind(t, pr3$age, pr3$sexe, pr3$inter, sint, cost)

### Direct estimation via mixtools
w0     <- 0.7
```

```
q0     <- 0.5
prova  <- regmixEM(pr3$incid, covars, lambda=c(w0, (1-w0)),
                   beta=matrix(c(mean(pr3$incid), 0, 0, 0, 0, 0, 0, mean(pr3$incid)/q0, 0, 0,
0, 0, 0, 0), ncol=2, nrow=ncol(covars)+1),
                   sigma=c(sd(pr3$incid), sd(pr3$incid)/q0),
                   k=2, addintercept=TRUE, epsilon=1e-16, maxit=10000)

### Initial values for covariates from linear regression model
linmod <- lm(pr3$incid~covars[, 1]+covars[, 2]+covars[, 3]+covars[, 4]+covars[, 5]+covars[,
6])

### Estimates, standard errors and confidence intervals for w and q
max.llh <- nlm(f=llh, p=c(log(prova$lambda[which(prova$beta[1,]==min(prova$beta[1,]))]/(1-
prova$lambda[which(prova$beta[1,]==min(prova$beta[1,]))])),
                          -0.5, linmod$coefficients,
prova$sigma[which(prova$beta[1,]==min(prova$beta[1,]))],
                          log((prova$beta[1,which(prova$beta[1,]==min(prova$beta[1,]))]/
                                 prova$beta[1,which(prova$beta[1,]==max(prova$beta[1,]))])/(1-
prova$beta[1,which(prova$beta[1,]==min(prova$beta[1,]))]/

prova$beta[1,which(prova$beta[1,]==max(prova$beta[1,]))]))),
               data=pr3$incid, covars=covars, hessian=TRUE)

q <- exp(max.llh$estimate[11])/(1+exp(max.llh$estimate[11]))

### Global validation (residuals analysis)
y_est <- vector()
w     <- vector()
for (i in 1:384)
{
  j <- (i %% 96)/96
  if (j == 0) j <- 1
  w[i] <-
exp(max.llh$estimate[1]+max.llh$estimate[2]*j)/(1+exp(max.llh$estimate[1]+max.llh$estimate[2]*
j))
  m    <- max.llh$estimate[3]+max.llh$estimate[4]*j+max.llh$estimate[5]*pr3$age[i]+

max.llh$estimate[6]*pr3$sexe[i]+max.llh$estimate[7]*pr3$inter[i]+max.llh$estimate[8]*covars[i,
5]+
    max.llh$estimate[9]*covars[i, 6]
  y_est[i] <- w[i]*m+(1-w[i])*m/q
}

y_est_agg_temp <- data.frame(t=rep(seq(1:96), 4), sexe=c(rep(0, 192), rep(1, 192)),
                             edat=c(rep(0, 96), rep(1, 96), rep(0, 96), rep(1, 96)), y_est)
tw             <- sum(unique(pr3$Pob))
y_est_agg      <- aggregate(y_est_agg_temp$y_est, by=list(y_est_agg_temp$t), FUN=sum)
y_agg          <- aggregate(pr3$incid, by=list(pr3$mes_any_problema), FUN=sum)
y_est_agg$x[1:12]  <- y_est_agg$x[1:12] *sum(unique(pr3$Pob[pr3$Year==2009]))/tw
y_est_agg$x[13:24] <- y_est_agg$x[13:24]*sum(unique(pr3$Pob[pr3$Year==2010]))/tw
y_est_agg$x[25:36] <- y_est_agg$x[25:36]*sum(unique(pr3$Pob[pr3$Year==2011]))/tw
y_est_agg$x[37:48] <- y_est_agg$x[37:48]*sum(unique(pr3$Pob[pr3$Year==2012]))/tw
y_est_agg$x[49:60] <- y_est_agg$x[49:60]*sum(unique(pr3$Pob[pr3$Year==2013]))/tw
y_est_agg$x[61:72] <- y_est_agg$x[61:72]*sum(unique(pr3$Pob[pr3$Year==2014]))/tw
y_est_agg$x[73:84] <- y_est_agg$x[73:84]*sum(unique(pr3$Pob[pr3$Year==2015]))/tw
y_est_agg$x[85:96] <- y_est_agg$x[85:96]*sum(unique(pr3$Pob[pr3$Year==2016]))/tw

y_agg$x[1:12]  <- y_agg$x[1:12] *sum(unique(pr3$Pob[pr3$Year==2009]))/tw
y_agg$x[13:24] <- y_agg$x[13:24]*sum(unique(pr3$Pob[pr3$Year==2010]))/tw
y_agg$x[25:36] <- y_agg$x[25:36]*sum(unique(pr3$Pob[pr3$Year==2011]))/tw
y_agg$x[37:48] <- y_agg$x[37:48]*sum(unique(pr3$Pob[pr3$Year==2012]))/tw
y_agg$x[49:60] <- y_agg$x[49:60]*sum(unique(pr3$Pob[pr3$Year==2013]))/tw
y_agg$x[61:72] <- y_agg$x[61:72]*sum(unique(pr3$Pob[pr3$Year==2014]))/tw
y_agg$x[73:84] <- y_agg$x[73:84]*sum(unique(pr3$Pob[pr3$Year==2015]))/tw
y_agg$x[85:96] <- y_agg$x[85:96]*sum(unique(pr3$Pob[pr3$Year==2016]))/tw

### Residuals
resid <- y_est_agg$x-y_agg$x

### ACF and PACF
bacf   <- acf(resid, lag.max = 10, plot = FALSE)
bacfdf <- with(bacf[1:10], data.frame(lag, acf))
conf.level <- 0.95
ciline <- qnorm((1 - conf.level)/2)/sqrt(length(y_agg$x))
q1 <- ggplot(data = bacfdf, mapping = aes(x = as.integer(lag), y = acf)) +
  geom_hline(aes(yintercept = 0)) + geom_hline(aes(yintercept = ciline), linetype=2) +
geom_hline(aes(yintercept = -ciline), linetype=2)+
```

```
  geom_segment(mapping = aes(xend = lag, yend = 0)) + ylab("") + xlab("Lag") + ggtitle("ACF")
+ theme(plot.title = element_text(hjust = 0.5))
bacf   <- pacf(resid, lag.max = 10, plot = FALSE)
bacfdf <- with(bacf, data.frame(lag, acf))
ciline <- qnorm((1 - conf.level)/2)/sqrt(length(y_agg$x))
q2 <- ggplot(data = bacfdf, mapping = aes(x = as.integer(lag), y = acf)) +
  geom_hline(aes(yintercept = 0)) + geom_hline(aes(yintercept = ciline), linetype=2) +
geom_hline(aes(yintercept = -ciline), linetype=2)+
  geom_segment(mapping = aes(xend = lag, yend = 0)) + ylab("") + xlab("Lag") + ggtitle("PACF")
+ theme(plot.title = element_text(hjust = 0.5))
grid.arrange(q1, q2, ncol=2)
```

4.4. Table 3 generation

```
### Construction of Table 3
library(mixtools)
source("R/llh_covars.R")

### Read the data
load("Data/data_CAT.RData")

### Covariates matrix
t         <- rep(seq(1, 96), 4)/96
pr4$inter <- as.numeric(pr4$age)*as.numeric(pr4$sexe)
sint      <- sin(2*pi*t/3)
cost      <- cos(2*pi*t/3)
covars    <- cbind(t, pr4$age, pr4$sexe, pr4$inter, sint, cost)

### Direct estimation via mixtools
w0     <- 0.7
q0     <- 0.5
prova  <- regmixEM(pr4$incid, covars, lambda=c(w0, (1-w0)),
                   beta=matrix(c(mean(pr4$incid), 0, 0, 0, 0, 0, 0, mean(pr4$incid)/q0, 0, 0,
0, 0, 0 ,0), ncol=2, nrow=ncol(covars)+1),
                   sigma=c(sd(pr4$incid), sd(pr4$incid)/q0),
                   k=2, addintercept=TRUE, epsilon=1e-16, maxit=10000)

### Initial values for covariates from linear regression model
linmod <- lm(pr4$incid~covars[, 1]+covars[, 2]+covars[, 3]+covars[, 4]+covars[, 5]+covars[,
6])

### Estimates, standard errors and confidence intervals for w and q
max.llh <- nlm(f=llh, p=c(log(prova$lambda[which(prova$beta[1,]==min(prova$beta[1,]))]/(1-
prova$lambda[which(prova$beta[1,]==min(prova$beta[1,]))])),
                          -0.5, linmod$coefficients,
prova$sigma[which(prova$beta[1,]==min(prova$beta[1,]))],
                          log((prova$beta[1,which(prova$beta[1,]==min(prova$beta[1,]))]/
                                 prova$beta[1,which(prova$beta[1,]==max(prova$beta[1,]))])/(1-
prova$beta[1,which(prova$beta[1,]==min(prova$beta[1,]))]/

prova$beta[1,which(prova$beta[1,]==max(prova$beta[1,]))]))),
               data=pr4$incid, covars=covars, hessian=TRUE)

q <- exp(max.llh$estimate[11])/(1+exp(max.llh$estimate[11]))

### Women 15-29
gw.women1 <- pr4[pr4$sexe==0 & pr4$age==0, ]
### Calculation of the posterior probabilities
post <- matrix(nrow=96, ncol=2)
w    <- vector()
for (i in 1:96)
{
  w[i] <-
exp(max.llh$estimate[1]+max.llh$estimate[2]*i/96)/(1+exp(max.llh$estimate[1]+max.llh$estimate[
2]*i/96))
  post[i, 1] <- w[i]*dnorm(gw.women1$incid[i],
mean=(max.llh$estimate[3]+max.llh$estimate[4]*i/96+max.llh$estimate[8]*covars[i, 5]+
                                                       max.llh$estimate[9]*covars[i, 6]),
sd=max.llh$estimate[10])/
    (w[i]*dnorm(gw.women1$incid[i],
mean=(max.llh$estimate[3]+max.llh$estimate[4]*i/96+max.llh$estimate[8]*covars[i, 5]+
                                            max.llh$estimate[9]*covars[i, 6]),
sd=max.llh$estimate[10])+
       (1-w[i])*dnorm(gw.women1$incid[i],
mean=(max.llh$estimate[3]+max.llh$estimate[4]*i/96+max.llh$estimate[8]*covars[i, 5]+
                                                    max.llh$estimate[9]*covars[i, 6])/q,
                      sd=max.llh$estimate[10]/q))
  post[i, 2] <- (1-w[i])*dnorm(gw.women1$incid[i],
mean=(max.llh$estimate[3]+max.llh$estimate[4]*i/96+max.llh$estimate[8]*covars[i, 5]+
```

```
                                                          max.llh$estimate[9]*covars[i,
6])/q, sd=max.llh$estimate[10]/q)/
    (w[i]*dnorm(gw.women1$incid[i],
mean=(max.llh$estimate[3]+max.llh$estimate[4]*i/96+max.llh$estimate[8]*covars[i, 5]+
                                           max.llh$estimate[9]*covars[i, 6]),
sd=max.llh$estimate[10])+
       (1-w[i])*dnorm(gw.women1$incid[i],
mean=(max.llh$estimate[3]+max.llh$estimate[4]*i/96+max.llh$estimate[8]*covars[i, 5]+
                                                 max.llh$estimate[9]*covars[i, 6])/q,
                      sd=max.llh$estimate[10]/q))
}
xrec <- ifelse(post[, 2] > 0.5, gw.women1$incid, gw.women1$incid/q)
gw.women1$xrec <- xrec

sum(gw.women1$N.GW)                              ### SIDIAP registered
round(sum(gw.women1$xrec*gw.women1$Pob/100000)) ### SIDIAP reconstructed
round(sum(gw.women1$incid*gw.women1$CatPop/100000)) ### Catalonia registered
round(sum(gw.women1$xrec*gw.women1$CatPop/100000))  ### Catalonia reconstructed

### Women 30-94
gw.women2 <- pr4[pr4$sexe==0 & pr4$age==1, ]
### Calculation of the posterior probabilities
post <- matrix(nrow=96, ncol=2)
w    <- vector()
for (i in 1:96)
{
  w[i] <-
exp(max.llh$estimate[1]+max.llh$estimate[2]*i/96)/(1+exp(max.llh$estimate[1]+max.llh$estimate[
2]*i/96))
  post[i, 1] <- w[i]*dnorm(gw.women2$incid[i],
mean=(max.llh$estimate[3]+max.llh$estimate[4]*i/96+max.llh$estimate[5]+
                                                      max.llh$estimate[8]*covars[i,
5]+max.llh$estimate[9]*covars[i, 6]), sd=max.llh$estimate[10])/
    (w[i]*dnorm(gw.women2$incid[i],
mean=(max.llh$estimate[3]+max.llh$estimate[4]*i/96+max.llh$estimate[5]+max.llh$estimate[8]*cov
ars[i, 5]+
                                           max.llh$estimate[9]*covars[i, 6]),
sd=max.llh$estimate[10])+
       (1-w[i])*dnorm(gw.women2$incid[i],
mean=(max.llh$estimate[3]+max.llh$estimate[4]*i/96+max.llh$estimate[5]+max.llh$estimate[8]*cov
ars[i, 5]+
                                                 max.llh$estimate[9]*covars[i, 6])/q,
                      sd=max.llh$estimate[10]/q))
  post[i, 2] <- (1-w[i])*dnorm(gw.women2$incid[i],
mean=(max.llh$estimate[3]+max.llh$estimate[4]*i/96+max.llh$estimate[5]+max.llh$estimate[8]*cov
ars[i, 5]+
                                                          max.llh$estimate[9]*covars[i,
6])/q, sd=max.llh$estimate[10]/q)/
    (w[i]*dnorm(gw.women2$incid[i],
mean=(max.llh$estimate[3]+max.llh$estimate[4]*i/96+max.llh$estimate[5]+max.llh$estimate[8]*cov
ars[i, 5]+
                                           max.llh$estimate[9]*covars[i, 6]),
sd=max.llh$estimate[10])+
       (1-w[i])*dnorm(gw.women2$incid[i],
mean=(max.llh$estimate[3]+max.llh$estimate[4]*i/96+max.llh$estimate[5]+max.llh$estimate[8]*cov
ars[i, 5]+
                                                 max.llh$estimate[9]*covars[i, 6])/q,
                      sd=max.llh$estimate[10]/q))
}
xrec <- ifelse(post[,2] > 0.5, gw.women2$incid, gw.women2$incid/q)
gw.women2$xrec <- xrec

sum(gw.women2$N.GW)                              ### SIDIAP registered
round(sum(gw.women2$xrec*gw.women2$Pob/100000)) ### SIDIAP reconstructed
round(sum(gw.women2$incid*gw.women2$CatPop/100000)) ### Catalonia registered
round(sum(gw.women2$xrec*gw.women2$CatPop/100000))  ### Catalonia reconstructed

### Men 15-29
gw.men1 <- pr4[pr4$sexe==1 & pr4$age==0, ]
### Calculation of the posterior probabilities
post <- matrix(nrow=96, ncol=2)
w    <- vector()
for (i in 1:96)
{
  w[i] <-
exp(max.llh$estimate[1]+max.llh$estimate[2]*i/96)/(1+exp(max.llh$estimate[1]+max.llh$estimate[
2]*i/96))
```

```
  post[i, 1] <- w[i]*dnorm(gw.men1$incid[i],
mean=(max.llh$estimate[3]+max.llh$estimate[4]*i/96+max.llh$estimate[6]+
                                                     max.llh$estimate[8]*covars[i,
5]+max.llh$estimate[9]*covars[i, 6]), sd=max.llh$estimate[10])/
     (w[i]*dnorm(gw.men1$incid[i],
mean=(max.llh$estimate[3]+max.llh$estimate[4]*i/96+max.llh$estimate[6]+max.llh$estimate[8]*cov
ars[i, 5]+
                                          max.llh$estimate[9]*covars[i, 6]),
sd=max.llh$estimate[10])+
       (1-w[i])*dnorm(gw.men1$incid[i],
mean=(max.llh$estimate[3]+max.llh$estimate[4]*i/96+max.llh$estimate[6]+max.llh$estimate[8]*cov
ars[i, 5]+
                                                max.llh$estimate[9]*covars[i, 6])/q,
                      sd=max.llh$estimate[10]/q))
  post[i, 2] <- (1-w[i])*dnorm(gw.men1$incid[i],
mean=(max.llh$estimate[3]+max.llh$estimate[4]*i/96+max.llh$estimate[6]+max.llh$estimate[8]*cov
ars[i, 5]+
                                                         max.llh$estimate[9]*covars[i, 6])/q,
sd=max.llh$estimate[10]/q)/
    (w[i]*dnorm(gw.men1$incid[i],
mean=(max.llh$estimate[3]+max.llh$estimate[4]*i/96+max.llh$estimate[6]+max.llh$estimate[8]*cov
ars[i, 5]+
                                          max.llh$estimate[9]*covars[i, 6]),
sd=max.llh$estimate[10])+
       (1-w[i])*dnorm(gw.men1$incid[i],
mean=(max.llh$estimate[3]+max.llh$estimate[4]*i/96+max.llh$estimate[6]+max.llh$estimate[8]*cov
ars[i, 5]+
                                                max.llh$estimate[9]*covars[i, 6])/q,
                      sd=max.llh$estimate[10]/q))
}
xrec <- ifelse(post[,2] > 0.5, gw.men1$incid, gw.men1$incid/q)
gw.men1$xrec <- xrec
sum(gw.men1$N.GW)                           ### SIDIAP registered
round(sum(gw.men1$xrec*gw.men1$Pob/100000)) ### SIDIAP reconstructed
round(sum(gw.men1$incid*gw.men1$CatPop/100000)) ### Catalonia registered
round(sum(gw.men1$xrec*gw.men1$CatPop/100000))  ### Catalonia reconstructed

### Men 30-94
gw.men2 <- pr4[pr4$sexe==1 & pr4$age==1, ]
### Calculation of the posterior probabilities
post <- matrix(nrow=96, ncol=2)
w    <- vector()
for (i in 1:96)
{
  w[i] <-
exp(max.llh$estimate[1]+max.llh$estimate[2]*i/96)/(1+exp(max.llh$estimate[1]+max.llh$estimate[
2]*i/96))
  post[i, 1] <- w[i]*dnorm(gw.men2$incid[i],
mean=(max.llh$estimate[3]+max.llh$estimate[4]*i/96+max.llh$estimate[5]+max.llh$estimate[6]+

max.llh$estimate[7]+max.llh$estimate[8]*covars[i, 5]+max.llh$estimate[9]*covars[i, 6]),
sd=max.llh$estimate[10])/
    (w[i]*dnorm(gw.men2$incid[i],
mean=(max.llh$estimate[3]+max.llh$estimate[4]*i/96+max.llh$estimate[5]+max.llh$estimate[6]+
                                          max.llh$estimate[7]+max.llh$estimate[8]*covars[i,
5]+max.llh$estimate[9]*covars[i, 6]), sd=max.llh$estimate[10])+
       (1-w[i])*dnorm(gw.men2$incid[i],
mean=(max.llh$estimate[3]+max.llh$estimate[4]*i/96+max.llh$estimate[5]+max.llh$estimate[6]+

max.llh$estimate[7]+max.llh$estimate[8]*covars[i, 5]+max.llh$estimate[9]*covars[i, 6])/q,
                      sd=max.llh$estimate[10]/q))
  post[i, 2] <- (1-w[i])*dnorm(gw.men2$incid[i],
mean=(max.llh$estimate[3]+max.llh$estimate[4]*i/96+max.llh$estimate[5]+max.llh$estimate[6]+

max.llh$estimate[7]+max.llh$estimate[8]*covars[i, 5]+max.llh$estimate[9]*covars[i, 6])/q,
sd=max.llh$estimate[10]/q)/
    (w[i]*dnorm(gw.men2$incid[i],
mean=(max.llh$estimate[3]+max.llh$estimate[4]*i/96+max.llh$estimate[5]+max.llh$estimate[6]+
                                          max.llh$estimate[7]+max.llh$estimate[8]*covars[i,
5]+max.llh$estimate[9]*covars[i, 6]), sd=max.llh$estimate[10])+
       (1-w[i])*dnorm(gw.men2$incid[i],
mean=(max.llh$estimate[3]+max.llh$estimate[4]*i/96+max.llh$estimate[5]+max.llh$estimate[6]+

max.llh$estimate[7]+max.llh$estimate[8]*covars[i, 5]+max.llh$estimate[9]*covars[i, 6])/q,
                      sd=max.llh$estimate[10]/q))
}
xrec <- ifelse(post[,2] > 0.5, gw.men2$incid, gw.men2$incid/q)
gw.men2$xrec <- xrec
```

```
sum(gw.men2$N.GW)                             ### SIDIAP registered
round(sum(gw.men2$xrec*gw.men2$Pob/100000)) ### SIDIAP reconstructed
round(sum(gw.men2$incid*gw.men2$CatPop/100000)) ### Catalonia registered
round(sum(gw.men2$xrec*gw.men2$CatPop/100000))  ### Catalonia reconstructed
```

4.5. Table S1 generation

```
### Construction of Table S1
library(gdata)
library(mixtools)
source("R/llh_covars.R")

### Read the data
dades <- read.xls("Data/GW_ICS_mes_any_edat_dec.xlsx", sheet=1)
vals  <- expand.grid(mes_any_problema = unique(dades$mes_any_problema),
                     sexe = unique(dades$sexe), edat_assig_dec = seq(0, 99, 1))
pr <- merge(vals, dades, all=TRUE)
rm(dades, vals)
pr$N.GW[is.na(pr$N.GW)] <- 0
pr$age_cat <- ifelse(pr$edat_assig_dec<=15, 0,
                     ifelse(pr$edat_assig_dec>15 & pr$edat_assig_dec<30, 1,
                            ifelse(pr$edat_assig_dec>=30 & pr$edat_assig_dec<99, 2, 3)))

pr2 <- aggregate(pr$N.GW, by=list(pr$mes_any_problema, pr$sexe, pr$age_cat), FUN=sum)
colnames(pr2) <- c("mes_any_problema", "sexe", "age", "N.GW")
pob <- read.xls("Data/GW_ICS_edat_dec.xlsx", sheet=2)
pob$age_cat <- ifelse(pob$edat_dec<=15, 0,
                      ifelse(pob$edat_dec>15 & pob$edat_dec<30, 1,
                             ifelse(pob$edat_dec>=30 & pob$edat_dec<99, 2, 3)))
pob <- aggregate(pob$N.poblacio.assignada..ICS., by=list(pob$periode, pob$sexe, pob$age_cat),
FUN=sum)
pr2$Year <- as.numeric(substr(pr2$mes_any_problema, 1, 4))
colnames(pob) <- c("Year", "sexe", "age", "Pob")
pr3 <- merge(pr2, pob, by=c("Year", "sexe", "age"))
pr3$incid <- pr3$N.GW/pr3$Pob*100000
rm(pr, pob, pr2)
pr3 <- pr3[order(pr3$sexe, pr3$age, pr3$mes_any_problema), ]
pr3$sexe2[pr3$sexe=="D"] <- 0
pr3$sexe2[pr3$sexe=="H"] <- 1
pr3$sexe <- NULL
colnames(pr3)[length(colnames(pr3))] <- "sexe"
catPop <- read.xls("Data/aec-253.xls")
catPop <- catPop[catPop$Age!="De 0 a 4 anys" & catPop$Age!="De 5 a 9 anys" &
                 catPop$Age!="De 10 a 14 anys", ]
catPop$AgeCat[catPop$Age=="De 15 a 19 anys" | catPop$Age=="De 20 a 24 anys" |
              catPop$Age=="De 25 a 29 anys"] <- 1
catPop$AgeCat[catPop$Age!="De 15 a 19 anys" & catPop$Age!="De 20 a 24 anys" &
              catPop$Age!="De 25 a 29 anys"] <- 2
catPop2 <- aggregate(catPop$Pop, by=list(catPop$AgeCat, catPop$Sex, catPop$Year), FUN=sum)
colnames(catPop2) <- c("age", "sexe", "Year", "CatPop")
pr4 <- merge(pr3, catPop2, by=c("age", "sexe", "Year"))
pr4 <- pr4[order(pr4$sexe, pr4$age, pr4$mes_any_problema), ]

### Remove < 15 years old
pr4 <- pr4[pr4$age>0 & pr4$age<3, ]

### Recode age group
pr4$age <- pr4$age-1

### Covariates matrix
t         <- rep(seq(1, 96), 4)/96
pr4$inter <- as.numeric(pr4$age)*as.numeric(pr4$sexe)
sint      <- sin(2*pi*t/3)
cost      <- cos(2*pi*t/3)
covars    <- cbind(t, pr4$age, pr4$sexe, pr4$inter, sint, cost)

### Direct estimation via mixtools (initial values provided by epidemiologists)
w0     <- 0.90
q0     <- 0.77
prova  <- regmixEM(pr4$incid, covars, lambda=c(w0, (1-w0)),
                   beta=matrix(c(mean(pr4$incid), 0, 0, 0, 0, 0, 0, mean(pr4$incid)/q0, 0, 0,
0, 0, 0 ,0), ncol=2, nrow=ncol(covars)+1),
                   sigma=c(sd(pr4$incid), sd(pr4$incid)/q0),
                   k=2, addintercept=TRUE, epsilon=1e-16, maxit=10000)

### Initial values for covariates from linear regression model
linmod <- lm(pr4$incid~covars[, 1]+covars[, 2]+covars[, 3]+covars[, 4]+covars[, 5]+covars[,
6])
```

```
### Estimates, standard errors and confidence intervals for w and q
max.llh <- nlm(f=llh, p=c(log(prova$lambda[which(prova$beta[1,]==min(prova$beta[1,]))]/(1-
prova$lambda[which(prova$beta[1,]==min(prova$beta[1,]))])),
                          -0.5, linmod$coefficients,
prova$sigma[which(prova$beta[1,]==min(prova$beta[1,]))],
                          log((prova$beta[1,which(prova$beta[1,]==min(prova$beta[1,]))]/
                                 prova$beta[1,which(prova$beta[1,]==max(prova$beta[1,]))])/(1-
prova$beta[1,which(prova$beta[1,]==min(prova$beta[1,]))]/

prova$beta[1,which(prova$beta[1,]==max(prova$beta[1,]))]))),
               data=pr4$incid, covars=covars, hessian=TRUE)

q <- exp(max.llh$estimate[11])/(1+exp(max.llh$estimate[11]))

### Women 16-29
gw.women1 <- pr4[pr4$sexe==0 & pr4$age==0, ]
### Calculation of the posterior probabilities
post <- matrix(nrow=96, ncol=2)
w    <- vector()
for (i in 1:96)
{
  w[i] <-
exp(max.llh$estimate[1]+max.llh$estimate[2]*i/96)/(1+exp(max.llh$estimate[1]+max.llh$estimate[
2]*i/96))
  post[i, 1] <- w[i]*dnorm(gw.women1$incid[i],
mean=(max.llh$estimate[3]+max.llh$estimate[4]*i/96+max.llh$estimate[8]*covars[i, 5]+
                                                       max.llh$estimate[9]*covars[i, 6]),
sd=max.llh$estimate[10])/
    (w[i]*dnorm(gw.women1$incid[i],
mean=(max.llh$estimate[3]+max.llh$estimate[4]*i/96+max.llh$estimate[8]*covars[i, 5]+
                                            max.llh$estimate[9]*covars[i, 6]),
sd=max.llh$estimate[10])+
       (1-w[i])*dnorm(gw.women1$incid[i],
mean=(max.llh$estimate[3]+max.llh$estimate[4]*i/96+max.llh$estimate[8]*covars[i, 5]+
                                                  max.llh$estimate[9]*covars[i, 6])/q,
                      sd=max.llh$estimate[10]/q))
  post[i, 2] <- (1-w[i])*dnorm(gw.women1$incid[i],
mean=(max.llh$estimate[3]+max.llh$estimate[4]*i/96+max.llh$estimate[8]*covars[i, 5]+
                                                           max.llh$estimate[9]*covars[i,
6])/q, sd=max.llh$estimate[10]/q)/
    (w[i]*dnorm(gw.women1$incid[i],
mean=(max.llh$estimate[3]+max.llh$estimate[4]*i/96+max.llh$estimate[8]*covars[i, 5]+
                                            max.llh$estimate[9]*covars[i, 6]),
sd=max.llh$estimate[10])+
       (1-w[i])*dnorm(gw.women1$incid[i],
mean=(max.llh$estimate[3]+max.llh$estimate[4]*i/96+max.llh$estimate[8]*covars[i, 5]+
                                                  max.llh$estimate[9]*covars[i, 6])/q,
                      sd=max.llh$estimate[10]/q))
}
xrec <- ifelse(post[, 2] > 0.5, gw.women1$incid, gw.women1$incid/q)
gw.women1$xrec <- xrec

gw.women1_ag <- aggregate(list(gw.women1$incid, gw.women1$xrec), by=list(gw.women1$Year),
FUN=mean)
colnames(gw.women1_ag) <- c("Year", "SIDIAP Reg", "SIDIAP Rec")
gw.women1_ag$Diff <- (gw.women1_ag$`SIDIAP Rec`-gw.women1_ag$`SIDIAP
Reg`)/gw.women1_ag$`SIDIAP Reg`*100
round(mean(gw.women1_ag$`SIDIAP Reg`), 2)
round(mean(gw.women1_ag$`SIDIAP Rec`), 2)
round((mean(gw.women1_ag$`SIDIAP Rec`)-mean(gw.women1_ag$`SIDIAP
Reg`))/mean(gw.women1_ag$`SIDIAP Reg`)*100, 2)

### Women >= 30
gw.women2 <- pr4[pr4$sexe==0 & pr4$age==1, ]
### Calculation of the posterior probabilities
post <- matrix(nrow=96, ncol=2)
w    <- vector()
for (i in 1:96)
{
  w[i] <-
exp(max.llh$estimate[1]+max.llh$estimate[2]*i/96)/(1+exp(max.llh$estimate[1]+max.llh$estimate[
2]*i/96))
  post[i, 1] <- w[i]*dnorm(gw.women2$incid[i],
mean=(max.llh$estimate[3]+max.llh$estimate[4]*i/96+max.llh$estimate[5]+
                                                       max.llh$estimate[8]*covars[i,
5]+max.llh$estimate[9]*covars[i, 6]), sd=max.llh$estimate[10])/
```

```
    (w[i]*dnorm(gw.women2$incid[i],
mean=(max.llh$estimate[3]+max.llh$estimate[4]*i/96+max.llh$estimate[5]+max.llh$estimate[8]*cov
ars[i, 5]+
                                            max.llh$estimate[9]*covars[i, 6]),
sd=max.llh$estimate[10])+
       (1-w[i])*dnorm(gw.women2$incid[i],
mean=(max.llh$estimate[3]+max.llh$estimate[4]*i/96+max.llh$estimate[5]+max.llh$estimate[8]*cov
ars[i, 5]+
                                                  max.llh$estimate[9]*covars[i, 6])/q,
                      sd=max.llh$estimate[10]/q))
  post[i, 2] <- (1-w[i])*dnorm(gw.women2$incid[i],
mean=(max.llh$estimate[3]+max.llh$estimate[4]*i/96+max.llh$estimate[5]+max.llh$estimate[8]*cov
ars[i, 5]+
                                                           max.llh$estimate[9]*covars[i,
6])/q, sd=max.llh$estimate[10]/q)/
    (w[i]*dnorm(gw.women2$incid[i],
mean=(max.llh$estimate[3]+max.llh$estimate[4]*i/96+max.llh$estimate[5]+max.llh$estimate[8]*cov
ars[i, 5]+
                                            max.llh$estimate[9]*covars[i, 6]),
sd=max.llh$estimate[10])+
       (1-w[i])*dnorm(gw.women2$incid[i],
mean=(max.llh$estimate[3]+max.llh$estimate[4]*i/96+max.llh$estimate[5]+max.llh$estimate[8]*cov
ars[i, 5]+
                                                  max.llh$estimate[9]*covars[i, 6])/q,
                      sd=max.llh$estimate[10]/q))
}
xrec <- ifelse(post[,2] > 0.5, gw.women2$incid, gw.women2$incid/q)
gw.women2$xrec <- xrec

gw.women2_ag <- aggregate(list(gw.women2$incid, gw.women2$xrec), by=list(gw.women2$Year),
FUN=mean)
colnames(gw.women2_ag) <- c("Year", "SIDIAP Reg", "SIDIAP Rec")
gw.women2_ag$Diff <- (gw.women2_ag$`SIDIAP Rec`-gw.women2_ag$`SIDIAP
Reg`)/gw.women2_ag$`SIDIAP Reg`*100
round(mean(gw.women2_ag$`SIDIAP Reg`), 2)
round(mean(gw.women2_ag$`SIDIAP Rec`), 2)
round((mean(gw.women2_ag$`SIDIAP Rec`)-mean(gw.women2_ag$`SIDIAP
Reg`))/mean(gw.women2_ag$`SIDIAP Reg`)*100, 2)

### Men 16-29
gw.men1 <- pr4[pr4$sexe==1 & pr4$age==0, ]
### Calculation of the posterior probabilities
post <- matrix(nrow=96, ncol=2)
w    <- vector()
for (i in 1:96)
{
  w[i] <-
exp(max.llh$estimate[1]+max.llh$estimate[2]*i/96)/(1+exp(max.llh$estimate[1]+max.llh$estimate[
2]*i/96))
  post[i, 1] <- w[i]*dnorm(gw.men1$incid[i],
mean=(max.llh$estimate[3]+max.llh$estimate[4]*i/96+max.llh$estimate[6]+
                                                     max.llh$estimate[8]*covars[i,
5]+max.llh$estimate[9]*covars[i, 6]), sd=max.llh$estimate[10])/
    (w[i]*dnorm(gw.men1$incid[i],
mean=(max.llh$estimate[3]+max.llh$estimate[4]*i/96+max.llh$estimate[6]+max.llh$estimate[8]*cov
ars[i, 5]+
                                          max.llh$estimate[9]*covars[i, 6]),
sd=max.llh$estimate[10])+
       (1-w[i])*dnorm(gw.men1$incid[i],
mean=(max.llh$estimate[3]+max.llh$estimate[4]*i/96+max.llh$estimate[6]+max.llh$estimate[8]*cov
ars[i, 5]+
                                                max.llh$estimate[9]*covars[i, 6])/q,
                      sd=max.llh$estimate[10]/q))
  post[i, 2] <- (1-w[i])*dnorm(gw.men1$incid[i],
mean=(max.llh$estimate[3]+max.llh$estimate[4]*i/96+max.llh$estimate[6]+max.llh$estimate[8]*cov
ars[i, 5]+
                                                         max.llh$estimate[9]*covars[i, 6])/q,
sd=max.llh$estimate[10]/q)/
    (w[i]*dnorm(gw.men1$incid[i],
mean=(max.llh$estimate[3]+max.llh$estimate[4]*i/96+max.llh$estimate[6]+max.llh$estimate[8]*cov
ars[i, 5]+
                                          max.llh$estimate[9]*covars[i, 6]),
sd=max.llh$estimate[10])+
       (1-w[i])*dnorm(gw.men1$incid[i],
mean=(max.llh$estimate[3]+max.llh$estimate[4]*i/96+max.llh$estimate[6]+max.llh$estimate[8]*cov
ars[i, 5]+
                                                max.llh$estimate[9]*covars[i, 6])/q,
                      sd=max.llh$estimate[10]/q))
```

```
}
xrec <- ifelse(post[,2] > 0.5, gw.men1$incid, gw.men1$incid/q)
gw.men1$xrec <- xrec
gw.men1_ag <- aggregate(list(gw.men1$incid, gw.men1$xrec), by=list(gw.men1$Year), FUN=mean)
colnames(gw.men1_ag) <- c("Year", "SIDIAP Reg", "SIDIAP Rec")
gw.men1_ag$Diff <- (gw.men1_ag$`SIDIAP Rec`-gw.men1_ag$`SIDIAP Reg`)/gw.men1_ag$`SIDIAP
Reg`*100
round(mean(gw.men1_ag$`SIDIAP Reg`), 2)
round(mean(gw.men1_ag$`SIDIAP Rec`), 2)
round((mean(gw.men1_ag$`SIDIAP Rec`)-mean(gw.men1_ag$`SIDIAP Reg`))/mean(gw.men1_ag$`SIDIAP
Reg`)*100, 2)

### Men >= 30
gw.men2 <- pr4[pr4$sexe==1 & pr4$age==1, ]
### Calculation of the posterior probabilities
post <- matrix(nrow=96, ncol=2)
w    <- vector()
for (i in 1:96)
{
  w[i] <-
exp(max.llh$estimate[1]+max.llh$estimate[2]*i/96)/(1+exp(max.llh$estimate[1]+max.llh$estimate[
2]*i/96))
  post[i, 1] <- w[i]*dnorm(gw.men2$incid[i],
mean=(max.llh$estimate[3]+max.llh$estimate[4]*i/96+max.llh$estimate[5]+max.llh$estimate[6]+

max.llh$estimate[7]+max.llh$estimate[8]*covars[i, 5]+max.llh$estimate[9]*covars[i, 6]),
sd=max.llh$estimate[10])/
    (w[i]*dnorm(gw.men2$incid[i],
mean=(max.llh$estimate[3]+max.llh$estimate[4]*i/96+max.llh$estimate[5]+max.llh$estimate[6]+
                                          max.llh$estimate[7]+max.llh$estimate[8]*covars[i,
5]+max.llh$estimate[9]*covars[i, 6]), sd=max.llh$estimate[10])+
       (1-w[i])*dnorm(gw.men2$incid[i],
mean=(max.llh$estimate[3]+max.llh$estimate[4]*i/96+max.llh$estimate[5]+max.llh$estimate[6]+

max.llh$estimate[7]+max.llh$estimate[8]*covars[i, 5]+max.llh$estimate[9]*covars[i, 6])/q,
                      sd=max.llh$estimate[10]/q))
  post[i, 2] <- (1-w[i])*dnorm(gw.men2$incid[i],
mean=(max.llh$estimate[3]+max.llh$estimate[4]*i/96+max.llh$estimate[5]+max.llh$estimate[6]+

max.llh$estimate[7]+max.llh$estimate[8]*covars[i, 5]+max.llh$estimate[9]*covars[i, 6])/q,
sd=max.llh$estimate[10]/q)/
    (w[i]*dnorm(gw.men2$incid[i],
mean=(max.llh$estimate[3]+max.llh$estimate[4]*i/96+max.llh$estimate[5]+max.llh$estimate[6]+
                                          max.llh$estimate[7]+max.llh$estimate[8]*covars[i,
5]+max.llh$estimate[9]*covars[i, 6]), sd=max.llh$estimate[10])+
       (1-w[i])*dnorm(gw.men2$incid[i],
mean=(max.llh$estimate[3]+max.llh$estimate[4]*i/96+max.llh$estimate[5]+max.llh$estimate[6]+

max.llh$estimate[7]+max.llh$estimate[8]*covars[i, 5]+max.llh$estimate[9]*covars[i, 6])/q,
                      sd=max.llh$estimate[10]/q))
}
xrec <- ifelse(post[,2] > 0.5, gw.men2$incid, gw.men2$incid/q)
gw.men2$xrec <- xrec
gw.men2_ag <- aggregate(list(gw.men2$incid, gw.men2$xrec), by=list(gw.men2$Year), FUN=mean)
colnames(gw.men2_ag) <- c("Year", "SIDIAP Reg", "SIDIAP Rec")
gw.men2_ag$Diff <- (gw.men2_ag$`SIDIAP Rec`-gw.men2_ag$`SIDIAP Reg`)/gw.men2_ag$`SIDIAP
Reg`*100
round(mean(gw.men2_ag$`SIDIAP Reg`), 2)
round(mean(gw.men2_ag$`SIDIAP Rec`), 2)
round((mean(gw.men2_ag$`SIDIAP Rec`)-mean(gw.men2_ag$`SIDIAP Reg`))/mean(gw.men2_ag$`SIDIAP
Reg`)*100, 2)
```
